# Proline‐Rich Transmembrane Protein 2 Is Variably Expressed Across Excitatory and Inhibitory Neurons in Mouse Motor Circuits

**DOI:** 10.1002/cne.70176

**Published:** 2026-06-04

**Authors:** Daisuke Hatta, Kaori Watanabe, Akira Kinoshita, Koh‐Ichiro Yoshiura, Naohiro Kurotaki, Keiro Shirotani, Nobuhisa Iwata

**Affiliations:** ^1^ Department of Genome‐based Drug Discovery, Graduate School of Biomedical Sciences Nagasaki University Nagasaki Japan; ^2^ Leading Medical Research Core Unit, Graduate School of Biomedical Sciences Nagasaki University Nagasaki Japan; ^3^ Department of Human Genetics, Atomic Bomb Disease Institute, Graduate School of Biomedical Sciences Nagasaki University Nagasaki Japan

**Keywords:** basal ganglia, dyskinesia, immunofluorescence, motor circuit, Prrt2

## Abstract

Proline‐rich transmembrane protein 2 (PRRT2) plays a pivotal role in the control of voluntary movements, as *PRRT2* mutations cause paroxysmal kinesigenic dyskinesia (PKD) in a loss‐of‐function manner. Although the cerebellum is considered a region responsible for PKD, we recently reported that Prrt2 also regulates dopaminergic activity in the striatum, suggesting that Prrt2 functions not only in the cerebellum but also in the basal ganglia motor circuits. However, the relationship between neuronal cell types expressing Prrt2 and motor functions remains poorly understood. In this study, we determined the neurochemical types of Prrt2‐positive neurons using immunofluorescence staining of mouse midbrain primary neurons and brain sections. Prrt2 was expressed mainly in glutamatergic and GABAergic neurons, but not in dopaminergic or cholinergic neurons. We found that Prrt2 was expressed preferentially in Vglut1‐positive, rather than Vglut2‐positive, cortical projection neurons and cerebellar granule cells, and in GABAergic medium spiny neurons of the basal ganglia, where Prrt2 was localized in axonal tracts and at or near presynaptic terminals. Taken together, we conclude that Prrt2 is variably expressed across excitatory and inhibitory neurons in motor‐related neural circuits, where it might play more diverse roles in the regulation of neuronal excitability and voluntary movement.

## Graphical abstract

Prrt2, a causal gene for paroxysmal kinesigenic dyskinesia, was sparsely expressed in nigrostriatal dopaminergic neurons, but alternatively in excitatory (within the cerebral cortex and cerebellum) or inhibitory (within the basal ganglia) neurons in a region‐dependent manner, suggesting that Prrt2 functions in both the basal ganglia and cerebellar loops.

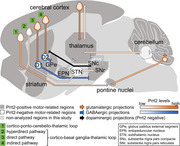

**Abbreviations**
EPN: entopeduncular nucleus
GPe: external globus pallidus
GPi: globus pallidus internal segment
KI: knock‐in
LFP: longitudinal fascicle of the pons
MCC: Manders’ correlation coefficient
PCC: Pearson's correlation coefficient
PKD: paroxysmal kinesigenic dyskinesia
Prrt2: proline‐rich transmembrane protein 2
SNc: substantia nigra pars compacta
SNr: substantia nigra pars reticulata
STN: subthalamic nucleus
VL: ventral lateral nucleus

## 1 Introduction

**FIGURE 1:**
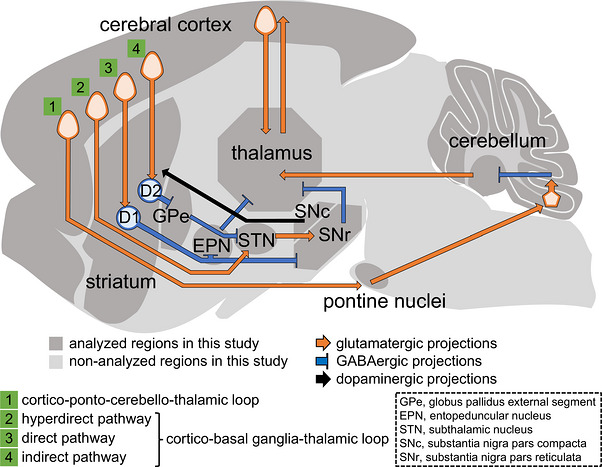
Motor circuits investigated in this study. The main motor circuits are illustrated in the schematic of the mouse brain. In this study, Prrt2 localization was analyzed in cortico‐ponto‐cerebello‐thalamic (1) and cortico‐basal ganglia‐thalamic loops (2–4), which consist of three pathways: the hyperdirect (2), direct (3), and indirect (4) pathways.

Proline‐rich transmembrane protein 2 (PRRT2) is an essential molecule for the control of voluntary movements, as loss of its function or expression causes paroxysmal kinesigenic dyskinesia (PKD) (Chen et al. 2011). Although Prrt2 roles in the cerebellum are responsible for PKD (Tan et al. 2018), we recently reported that *Prrt2* knock‐in (KI) mice harboring a PKD‐related *Prrt2* mutation showed dopamine‐related motor deficits (Hatta et al. 2023) and excessive dopamine release in the striatum (Hatta et al. 2024). Therefore, Prrt2 is important for controlling both of the main motor circuits: the cortico‐basal ganglia‐thalamic and cortico‐ponto‐cerebello‐thalamic loops (Li et al. 2023) (Figure 1). The former loop is regulated by nigrostriatal dopaminergic neurons from the substantia nigra pars compacta (SNc) and is separated into three pathways: the direct, indirect, and hyperdirect pathways (Chiken et al. 2015). The direct pathway passes through the striatum and substantia nigra pars reticulata (SNr) or entopeduncular nucleus (EPN, a rodent homolog of the globus pallidus internal segment, GPi) in the basal ganglia. The indirect pathway connects the striatum to the SNr or EPN via the external globus pallidus (GPe) and subthalamic nucleus (STN). The hyperdirect pathway passes through the STN and SNr or EPN in the basal ganglia. In the latter loop, nerve tracts pass through the pontine nuclei and cerebellum between the cerebral cortex and thalamus (Kratochwil et al. 2017). Although it has been analyzed using fluorescent immunocytochemistry of the primary neurons (Ferrante et al. 2021; Savino et al. 2021; Sterlini et al. 2021; Valente et al. 2019) and immunohistochemistry (Hatta et al. 2023; Tan et al. 2018), *Prrt2* promoter‐driven β‐gal staining (Calame et al. 2020; Michetti et al. 2017), or in situ hybridization (Chen et al. 2011) of mouse brain sections, it has not been determined what kinds of neurons in the loops express Prrt2. To deeply understand the relationship between Prrt2 and motor control, detailed characterization of Prrt2‐positive neuronal cell types is required. In this study, we prepared mouse midbrain primary cultures, which contain dopaminergic neurons, and brain sections, and comprehensively investigated the neurochemical types of Prrt2‐expressing neurons by immunofluorescence staining and subsequent colocalization analyses between Prrt2 and several neurochemical type‐ or region‐specific markers: Vglut1 and Vglut2 for glutamatergic neurons; vesicular GABA transporter (Vgat), glutamate decarboxylase 65 (Gad65), and Gad67 for GABAergic neurons; tyrosine hydroxylase (Th) and dopamine transporter (Dat) for dopaminergic neurons; and dopamine‐ and cAMP‐regulated phosphoprotein, Mr 32 kDa (Darpp‐32) for striatal medium spiny neurons.

## 2 Materials and Methods

### 2.1 Animals

All animal experiments in this study were approved by the Institutional Animal Care and Use Committee of Nagasaki University and were conducted in accordance with the Guidelines for Animal Experimentation of Nagasaki University. In the present study, we used wild‐type and *Prrt2* KI mice (Hatta et al. 2023) on a C57BL/6J background to obtain adult mouse brains or primary midbrain neurons. The adult mouse brains were excised after transcardial perfusion with chilled 0.1 M phosphate‐buffered saline (PBS; pH 7.4) under three‐drug‐mixed anesthesia by intraperitoneal administration of a 6 mL/kg mixture of 0.5 mg/mL butorphanol tartrate (3 mg/kg, Vetorphale, Meiji Seika Pharma, Tokyo, Japan), 0.15 mg/mL medetomidine hydrochloride (0.45 mg/kg, Dorbene vet, Kyoritsu Seiyaku, Tokyo, Japan), and 0.8 mg/mL midazolam (2.4 mg/kg, Sandoz K.K., Tokyo, Japan).

### 2.2 Preparation of Mouse Primary Midbrain Neurons

Midbrain primary neurons were prepared from E13 wild‐type or *Prrt2* KI mouse brains as previously described (Gaven et al. 2014), with some modifications (Hatta et al. 2020), and cultured in Neurobasal medium (Thermo Fisher Scientific, Inc., Waltham, MA, USA) supplemented with 2% B‐27 supplement (Thermo Fisher Scientific, Inc.), 0.5 mM glutamine, 100 U/mL penicillin, and 100 µg/mL streptomycin on poly L‐lysine‐coated cover glasses in a humidified atmosphere of 5% CO_2_ at 37°C.

### 2.3 Fluorescent Immunocytochemistry

Mouse midbrain primary neurons on cover glasses were fixed with 4% paraformaldehyde in 0.1 M phosphate buffer (pH 7.4) for 10 min at room temperature and autoclaved in citrate buffer (pH 6.0) at 120°C for 5 min. Neurons were permeabilized with 0.2% Triton X‐100/PBS for 10 min and blocked with Tris‐NaCl‐blocking (TNB) buffer (FP1012, PerkinElmer, Waltham, MA, USA) for 1 h. Subsequently, neurons were probed with primary antibodies at 4°C overnight, followed by incubation with secondary antibodies at room temperature for 1 h. In double or triple staining, this procedure was repeated to label another molecule, and the sections were finally mounted in mounting medium (H‐2000‐2, Vector Laboratories, Inc., Burlingame, CA, USA). Information on the antibodies used for immunocytochemistry is provided in Tables 1 and 2. Images were acquired with a 63× objective using a confocal microscope (LSM710, RRID:SCR_018063, Carl Zeiss, Oberkochen, Germany).

**TABLE 1:** List of primary antibodies used in this study.

Name of antibodies	Host species	Dilution	Catalog #, supplier, and RRID
Anti‐Prrt2	Rabbit	1:400 for ICC 1:800 for IHC	HPA014447, Sigma‐Aldrich, Saint Louis, MO, USA, RRID:AB_1855786
Anti‐Vglut1	Mouse	1:150 for ICC	MAB5502, Merck Millipore, Darmstadt, Germany, RRID:AB_11214451
Anti‐Vglut1	Mouse	1:2000 for IHC	sc‐377425, Santa Cruz Biotechnology, Inc., Dallas, TX, USA, RRID: AB_2687960
Anti‐Vglut2	Mouse	1:250 for ICC 1:2500 for IHC	MAB5504, Merck Millipore, RRID:AB_2187552
Anti‐Vgat	Mouse	1:150 for ICC 1:500 for IHC	sc‐393373, Santa Cruz Biotechnology, Inc., RRID:AB_2801273
Anti‐Vgat	Rabbit	1:2000 for IHC	131 002; Synaptic Systems GmbH, Göttingen, Germany, RRID:AB_887871
Anti‐Gad65	Mouse	1:150 for ICC	sc‐377145, Santa Cruz Biotechnology, Inc., RRID:AB_2619725
Anti‐Gad67	Mouse	1:150 for ICC	sc‐28376, Santa Cruz Biotechnology, Inc., RRID:AB_627650
Anti‐Darpp‐32	Mouse	1:10,000 for IHC	sc‐271111, Santa Cruz Biotechnology, Inc., RRID:AB_10610055
Anti‐Dat	Rat	1:120 for ICC 1:300 for IHC	sc‐32258, Santa Cruz Biotechnology, Inc., RRID:AB_627400
Anti‐Th	Mouse	1:120 for ICC	sc‐25269, Santa Cruz Biotechnology, Inc., RRID:AB_628422
Anti‐Chat	Goat	1:100 for IHC	AB144P, Merck Millipore, RRID:AB_2079751
Anti‐Stx1a	Mouse	1:3000 for IHC	S0664, Sigma‐Aldrich, RRID:AB_477483
Anti‐Stx1a	Rabbit	1:50 for IHC	574784, Merck Millipore, RRID:AB _2198508
Anti‐Vamp2	Mouse	1:200 for IHC	sc‐69706, Santa Cruz Biotechnology, Inc., RRID:AB_2212614
Anti‐Vamp2	Rabbit	1:800 for IHC	ANR‐007, Alomone Labs, RRID:AB_2040220

Abbreviations: ICC, fluorescent immunocytochemistry; IHC: fluorescent immunohistochemistry.

**TABLE 2:** List of secondary antibodies and amplification reagents used in this study.

Name of antibodies or reagents	Host species	Dilution or concentration	Catalog #, supplier, and RRID
HRP polymer‐linked anti‐rabbit IgG	Goat	Undiluted for IHC	K4003, Dako, Glostrup, Denmark, RRID:AB_2630375
HRP polymer‐linked anti‐mouse IgG	Goat	Undiluted for IHC	K4001, Dako, RRID: AB_2827819
HRP‐linked anti‐rat IgG	Rabbit	1:600 for IHC	7077, Cell Signaling Technology, Danvers, MA, USA RRID:AB_10694715
Alexa 568‐linked anti‐goat IgG	Donkey	1:500 for IHC	A‐11057, Molecular Probes, Eugene, OR, USA RRID:AB_142581
Alexa 488‐linked anti‐rat IgG	Goat	1:500 for ICC	A‐11006, Molecular Probes, RRID:AB_141373
Alexa 568‐linked anti‐rabbit IgG	Goat	1:500 for ICC	A‐11036, Molecular Probes, RRID:AB_10563566
Alexa 647‐linked anti‐mouse IgG	Goat	1:500 for ICC	A‐32728, Molecular Probes, RRID:AB_2633277
FITC‐linked tyramide	—	1:50 for IHC	SAT701001EA, Akoya Biosciences, Inc., Marlborough, MA, USA
Cy5‐linked tyramide	—	1:50 for IHC	SAT705A001EA, Akoya Biosciences, Inc.
Cy5‐linked tyramide	—	25 µM for IHC	11066, AAT Bioquest, Inc., Pleasanton, CA, USA
Cy3‐linked tyramide	—	1:50 for IHC	SAT704A001EA, Akoya Biosciences, Inc.
Cy3‐linked tyramide	—	20 µM for IHC	11065, AAT Bioquest, Inc.

Abbreviations: ICC, fluorescent immunocytochemistry, IHC: fluorescent immunohistochemistry.

### 2.4 Fluorescent Immunohistochemistry

Brain sagittal sections from 11‐ to 15‐week‐old male mice were immunostained with the antibodies listed in Tables 1 and 2 based on previously reported protocols (Fukami et al. 2002; Hatta et al. 2023). Briefly, mouse brains were fixed with 4% paraformaldehyde in 0.1 M PB (pH 7.4) at 4°C overnight and then transferred to PBS, followed by paraffin embedding and sectioning at a thickness of 5 µm using a rotary microtome (HM 355S, RRID:SCR_026146, Thermo Fisher Scientific, Inc.). The sagittal sections were then adhered to glass slides. After deparaffinization using xylene and ethanol, the sections (1.0–1.7 mm lateral from the midline) were autoclaved at 120°C for 5 min in Tris‐EDTA buffer (pH 9.0) and then soaked in methanol containing 0.3% hydrogen peroxide for 30 min and subsequently in TNB buffer for 60 min at room temperature. Primary antibodies, secondary antibodies (horseradish peroxidase‐linked anti‐IgG antibodies), and fluorophore‐conjugated tyramide with 0.003% hydrogen peroxide were applied to the sections at 4°C overnight, at room temperature for 1 h, and at room temperature for 10 min, respectively. After each incubation, sections were washed with Tris‐NaCl‐Tween (TNT) buffer (0.1 M Tris‐HCl, 0.15 M NaCl, and 0.05% Tween 20). In the case of multiple immunostaining, after sections were subjected to treatment with 3% hydrogen peroxide in PBS for 15 min and blocked with TNB buffer for 60 min, the second or third primary antibodies and subsequent incubations were performed using the same procedure. After immunostaining, the sections were mounted in mounting medium (TA‐030‐FM, Thermo Fisher Scientific, Inc.). Images were acquired using a 20× objective on a digital slide scanner (Nanozoomer S60, C13210, RRID:SCR_022537, Hamamatsu Photonics, Shizuoka, Japan), a 4× objective on a fluorescence microscope (BZ‐9000, RRID:SCR_015486, Keyence, Osaka, Japan), or a 10× or 100× objective on a confocal microscope (LSM710, RRID:SCR_018063, or LSM800, RRID:SCR_015963, Carl Zeiss).

### 2.5 Image Analysis

All image analyses were performed using ImageJ Fiji software (Schindelin et al. 2012, RRID:SCR_002285). Mean pixel intensity of Prrt2 or marker proteins in each brain region was quantified via a “measure” function in Fiji. Quantitative colocalization analysis was performed using the JACoP plugin (Bolte and Cordelières 2006, RRID:SCR_025164) in Fiji. For the immunocytochemistry images, an object‐based colocalization analysis was applied because most immunosignals were detected in a punctate pattern, in which the proportion of Prrt2‐colocalizing puncta in the marker puncta number was calculated. Pixel‐based colocalization analysis was applied to the fluorescent immunohistochemistry images, in which the Manders’ correlation coefficient (MCC) and Pearson's correlation coefficient (PCC) between Prrt2 and marker proteins were computed. All immunohistochemistry images were subjected to rolling‐ball background subtraction (Pike et al. 2017) and equivalent thresholding before colocalization analyses.

### 2.6 Statistical Analyses

All quantitative data in this study are expressed as mean ± standard error. Statistical analyses were conducted using SigmaPlot software ver.14.0 (RRID:SCR_003210, Systat Software Inc., San Jose, CA, USA). For comparisons of the means between two groups, statistical analysis was performed using Student's *t*‐test. One‐way or two‐way analysis of variance (ANOVA) was performed to compare the means among three or more groups, followed by a post hoc Bonferroni test. Differences were considered significant when *p* values were less than 0.05.

## 3 Results

### 3.1 Prrt2 Is Expressed in Glutamatergic and GABAergic Neurons Rather Than in Dopaminergic Neurons in Midbrain Primary Culture

**FIGURE 2:**
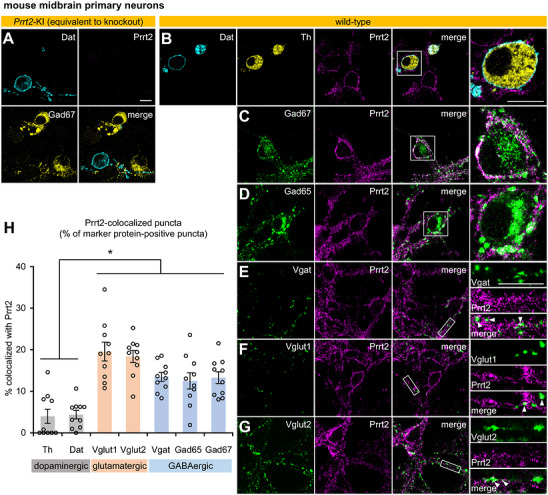
Neurochemical types of Prrt2‐expressing neurons in mouse midbrain primary culture. (A–G) Representative immunofluorescence images of mouse midbrain primary neurons obtained using a confocal microscope with a 63× objective. Scale bar, 100 µm. As an experimental control for Prrt2 immunoreactivity, neurons from *Prrt2* KI mice harboring a PKD‐related mutation that abolishes Prrt2 expression were co‐immunostained with anti‐Prrt2, anti‐dopamine transporter (Dat), and anti‐glutamate decarboxylase 67 (Gad67) antibodies (A). Neurons from wild‐type mice were immunostained with anti‐Prrt2 antibodies together with antibodies against several neurochemical marker proteins: Dat + tyrosine hydroxylase (Th) (B), Gad67 (C), Gad65 (D), vesicular GABA transporter (Vgat) (E), vesicular glutamate transporter 1 (Vglut1) (F), and Vglut2 (G). The right panels show a magnified view of the boxed area in merged images (B–G). Arrowheads indicate partially overlapping puncta. (H) The proportion of Prrt2‐colocalized puncta to total puncta for each marker protein. Data are expressed as the mean ± SE and individual value plots (*n* = 10 fields from two experiments, 5 fields/experiment). One‐way ANOVA: *F*_(6, 63)_ = 14.30, *p* < 0.001; Bonferroni post hoc test: Th vs. Dat, *p* = 1.000; Th vs. Vglut1 or Vglut2, *p* < 0.001; Th vs. Vgat, *p* = 0.002; Th vs. Gad65, *p* = 0.009; Th vs. Gad67, *p* = 0.003; Dat vs. Vglut1 or Vglut2, *p* < 0.001; Dat vs. Vgat, *p* = 0.004; Dat vs. Gad65, *p* = 0.015; Dat vs. Gad67, *p* = 0.005. *p < 0.05.

To clarify the neurochemical types of Prrt2‐expressing neurons, we performed immunofluorescence staining of the midbrain primary neurons to co‐label Prrt2 and marker proteins (Figure 2). We first confirmed that Prrt2 was specifically detected in the wild‐type (Figure 2B–G) but not in *Prrt2* KI neurons (Figure 2A). The *Prrt2* KI mice harbor a PKD‐associated mutation that abolishes detectable Prrt2 protein expression by immunostaining, rendering them functionally equivalent to the knock‐out model (Hatta et al. 2023). Prrt2 was localized to the somatic membrane and neurites (Figure 2B), which did not colocalize with the neurites of Th‐ and Dat‐positive neurons (Figure 2B); however, neurites from adjacent Prrt2‐positive neurons surrounded dopaminergic neurons (Figure 2B), suggesting that Prrt2 controls the activity of dopaminergic neurons indirectly via neural transmission, as we previously showed (Hatta et al. 2024). The cytoplasmic GABAergic markers, Gad67 (Figure 2C) and Gad65 (Figure 2D), were expressed in the soma of Prrt2‐positive neurons. A presynaptic GABAergic marker, Vgat, showed partial colocalization with Prrt2 (Figure 2E). Similarly, the presynaptic glutamatergic markers, Vglut1 (Figure 2F) and Vglut2 (Figure 2G), partially overlapped with Prrt2‐positive axons. Colocalization analysis showed that Prrt2 existed more frequently in glutamatergic and GABAergic neurons than in dopaminergic neurons (*p* < 0.05) (Figure 2H). However, since the colocalization of Prrt2 with presynaptic markers, Vglut1, Vglut2, or Vgat, was observed in only a small fraction (Figure 2E–H), Prrt2 might not be exclusively enriched at presynaptic sites, but in various subcellular locations, including the somatic membrane, axons, and perisynaptic areas.

### 3.2 Prrt2 Is Expressed in Different Types of Neurons in a Region‐Specific Manner

**FIGURE 3:**
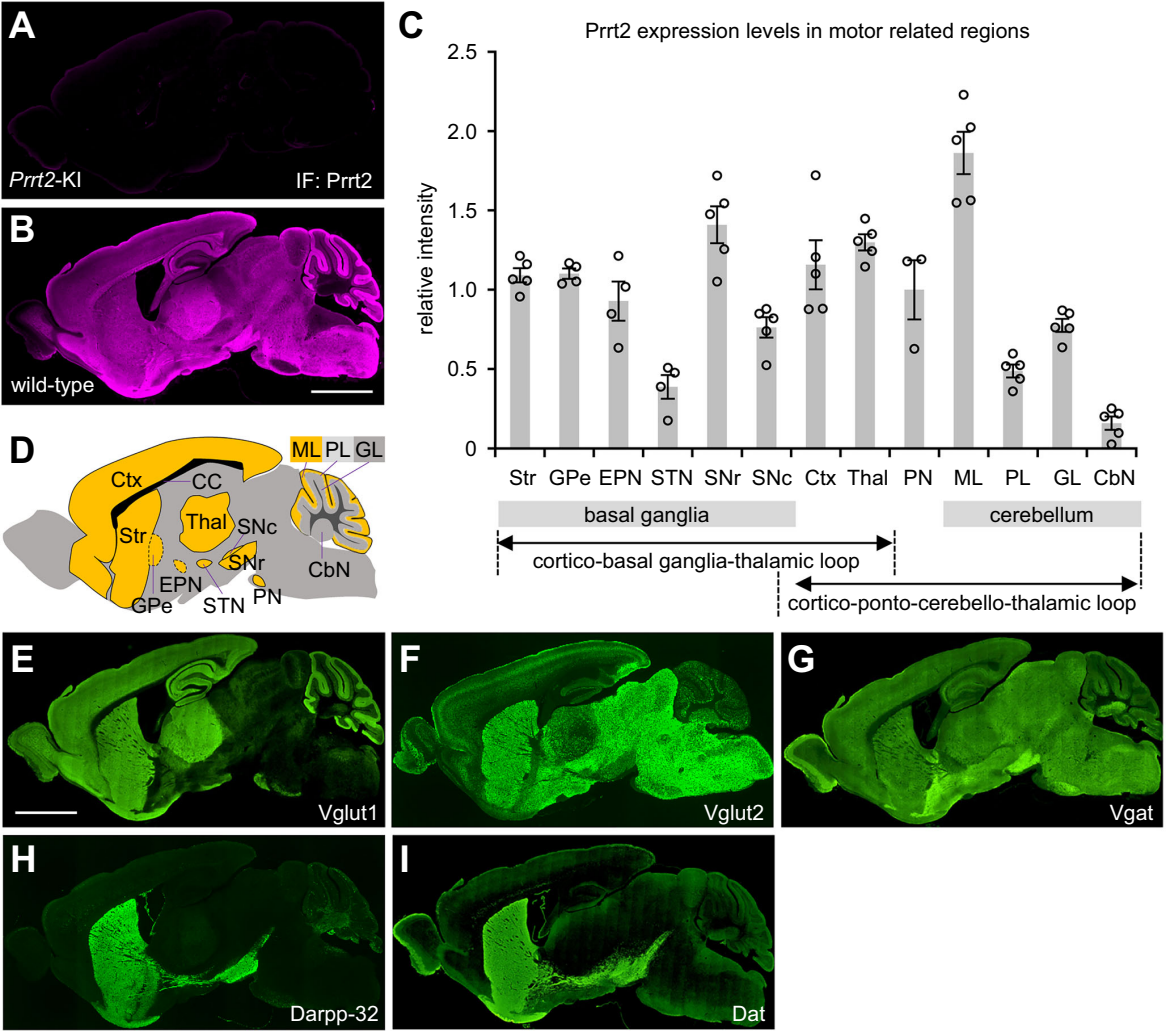
Prrt2 expression patterns in the mouse whole brain. (A, B) Sagittal brain sections from *Prrt2* KI mice carrying a PKD‐related mutation that leads to the absence of Prrt2 expression (A) and from wild‐type mice (B) were immunostained with an anti‐PRRT2 antibody and imaged using a fluorescence microscope at low power. (C) Prrt2 intensities in motor‐related brain regions of wild‐type mice were quantified. Data are expressed as mean ± SE and individual value plots of *n* = 3–5 mice. (D) Schematic sagittal brain section showing the positions of the brain regions analyzed in panel C. The regions surrounded by dashed lines represent areas that are not contained in the Prrt2‐stained section B. (E–I) Sagittal brain sections of wild‐type mice were immunostained with antibodies against several marker molecules: Vglut1 (E), Vglut2 (F), Vgat (G), Darpp‐32 (H), and Dat (I), and the whole brains were imaged using a fluorescence microscope. Scale bars, 2 mm. Str, striatum; GPe, globus pallidus external segment; EPN, entopeduncular nucleus; STN, subthalamic nucleus; SNr, substantia nigra pars reticulata; SNc, substantia nigra pars compacta; Ctx, cerebral cortex; Thal, thalamus; PN, pontine nuclei; ML, molecular layer; PL, Purkinje cell layer; GL, granular layer; CbN, cerebellar nuclei; CC, corpus callosum.

To elucidate the regional expression pattern of Prrt2 in the brain, we performed immunofluorescence staining of mouse brain sections and obtained images of the whole brain (Figure 3) or each region (Figures 4, 5, 6), followed by colocalization analysis between Prrt2 and marker proteins (Figure 6). Prrt2 immunosignals in wild‐type mouse brain were specific (Figure 3B), because it was not detected in *Prrt2* KI mouse brain (Figure 3A). The specificity of this antibody has also been demonstrated by western blotting in wild‐type and *Prrt2* KI mouse brains (Hatta et al., 2023), showing a clear single band in wild‐type mouse brains but not in *Prrt2* KI brains. To ensure the reliability of the specific Prrt2 staining, we tested several other commercially available and our own antibodies (Hatta et al. 2023) targeting different epitopes, but only the antibody used in this study yielded specific staining suitable for immunohistochemical analysis. However, our staining methods are well established, as previously described (Fukami et al. 2002), and the Prrt2 staining pattern (Figure 3B) is consistent with that reported by other institutions using a noncommercial antibody targeting a different epitope (Tan et al. 2018), supporting the specificity and reliability of our Prrt2 immunostaining.

FIGURE 4Prrt2 expression patterns in the cerebral cortex, thalamus, pons, and cerebellum. Mouse sagittal brain sections were labeled with anti‐Prrt2 together with anti‐Vglut1 (A1, B1, C1, D1, E1, F1, G1, H1, I1), anti‐Vglut2 (A2, B2, C2, D2, E2, F2, G2, H2, I2), or anti‐Vgat (A3, B3, C3, D3, E3, F3, G3, H3, I3) antibodies. The cerebral cortex (A, E), thalamus (B, F), pontine nuclei (G) in the ventral pons (C), and molecular (H) and granular (I) layers of the cerebellum (D) were imaged using a confocal microscope with a 10× (A–D) or 100× (E–I) objective. Scale bars, 200 µm (A–D) and 20 µm (E–I). The numbers I–VI in panel A represent layers of the cerebral cortex. Rt, thalamic reticular nucleus; VL, ventral lateral nucleus; VPM, ventral posteromedial nucleus; VPL, ventral posterolateral Nucleus; PN, pontine nuclei; LFP, longitudinal fascicle of the pons; PO, periolivary region; ML, molecular layer; PL, Purkinje cell layer; GL, granular layer; CbN, cerebellar nuclei.

**Figure:**
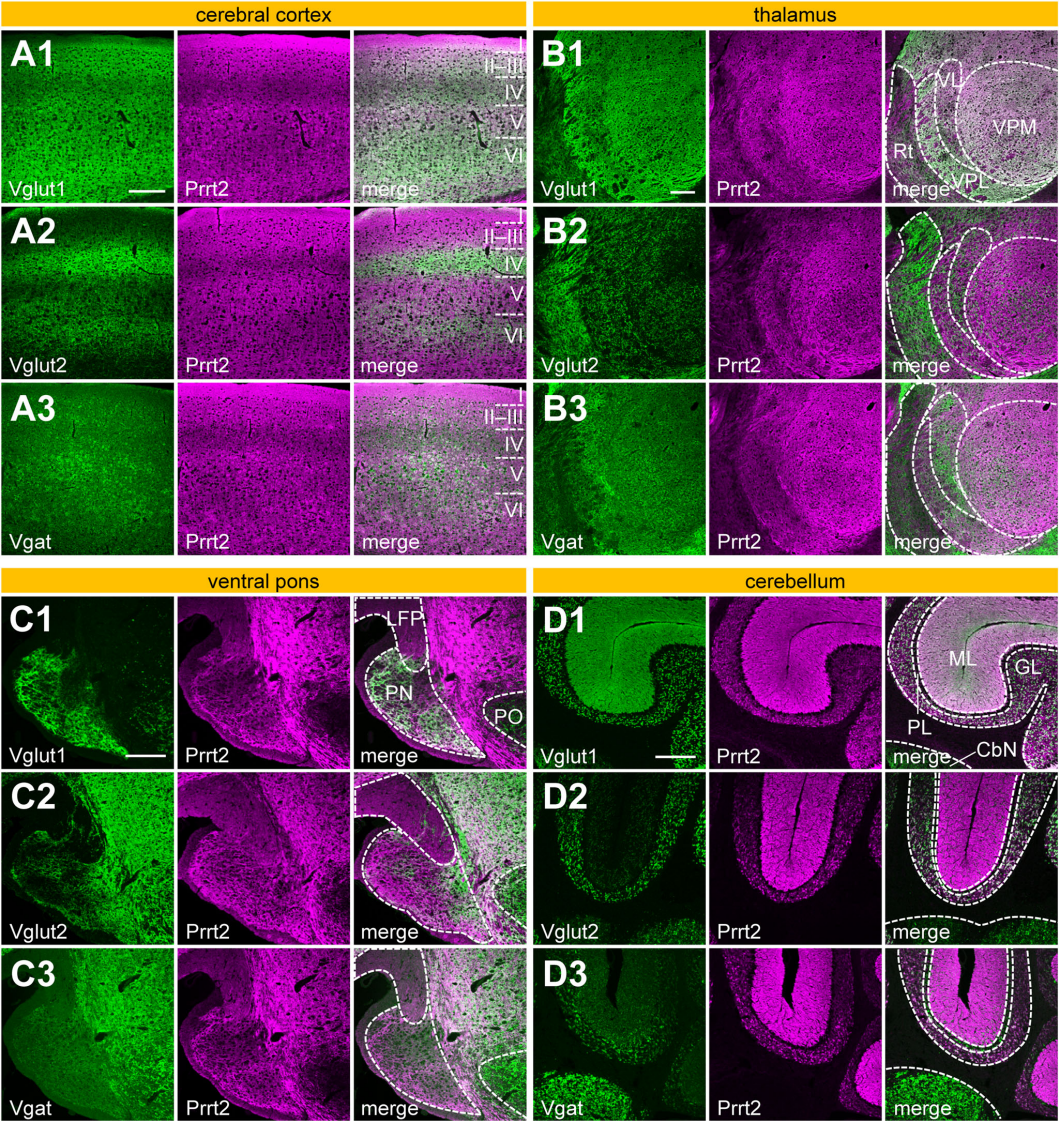

**Figure:**
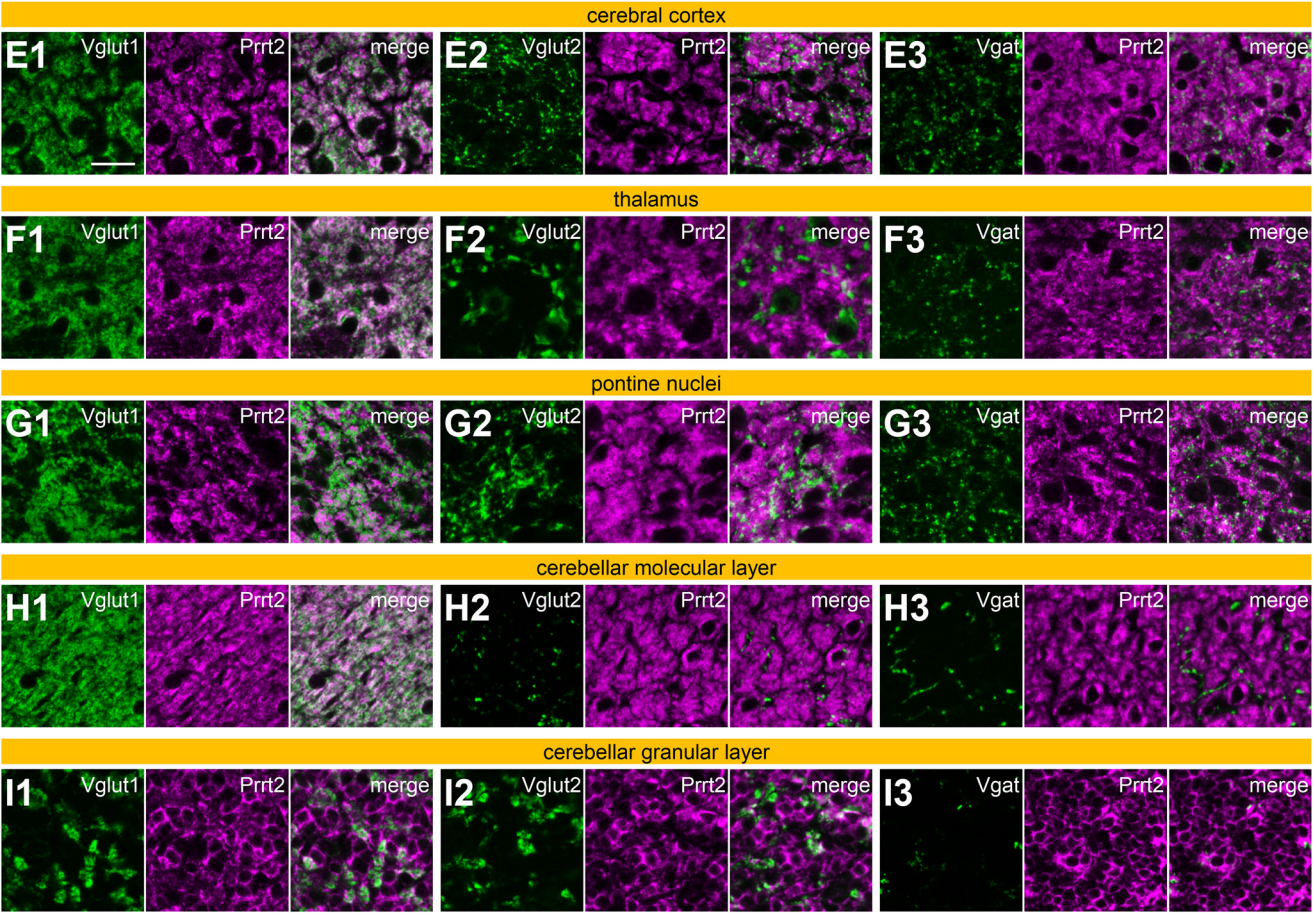

FIGURE 5Prrt2 expression patterns in the mouse basal ganglia. (A) Sagittal brain sections of wild‐type mice were co‐immunostained with anti‐Darpp‐32 and anti‐PRRT2 antibodies, and the basal ganglia were imaged using a fluorescence microscope at low power. Boxes in the right panel (merge) show areas for the following (B–D) higher‐powered imaging. (B–I) Mouse sagittal brain sections were immunostained with anti‐PRRT2 antibodies together with antibodies against Vglut1 (B1, C1, D1, E1), Vglut2 (B2, C2, D2, E2, F1, G1, H1, I1), Vgat (B3, C3, D3, E3, F2, G2, H2, I2), Darpp‐32 (B4, C4, D4, E4, F3, G3, H3), Dat (B5, C5, D5, E5, F4, G4, H4, I3), or Chat (A6). The striatum (Str) + external globus pallidus (GPe) (B), entopeduncular nucleus (EPN) + subthalamic nucleus (STN) (C), and substantia nigra pars reticulata (SNr) + compacta (SNc) (D) were imaged using a confocal microscope with a 10× objective lens. The striatum (E), GPe (F), EPN (G), SNr (H), and SNc (I) were imaged using a confocal microscope with a 100× objective. (J–L) Wild‐type brain sections were co‐immunostained with anti‐Dat, anti‐Darpp‐32, and anti‐Prrt2 antibodies, and the EPN (rectangular area in J) was imaged using a confocal microscope with a 100× objective (K). The nigrostriatal dopaminergic (Dat‐positive) and striatonigral GABAergic projections (Darpp‐32‐positive) are surrounded by dotted and solid lines, respectively (K). Manders’ correlation coefficient (MCC, intensity ratio of marker protein‐overlapping Prrt2 to total Prrt2) and Pearson's correlation coefficient (PCC, correlation of localization patterns between Prrt2 and marker proteins) between Prrt2 and Dat or Darpp‐32 in the axonal areas of the above projections were calculated (L). Data are expressed as the mean ± SE and individual value plots of *n* = 24 Dat‐positive axons and *n* = 17 Darpp‐32‐positive axons from four mice. Student's *t*‐test: *t*_(39)_ = −12.769, *p* < 0.001 (MCC, left); *t*_(39)_ = −17.031, *p* < 0.001 (PCC, right). *p < 0.05. Scale bar, 2 mm (A), 200 µm (B–D), and 20 µm (E–I, K).

**Figure:**
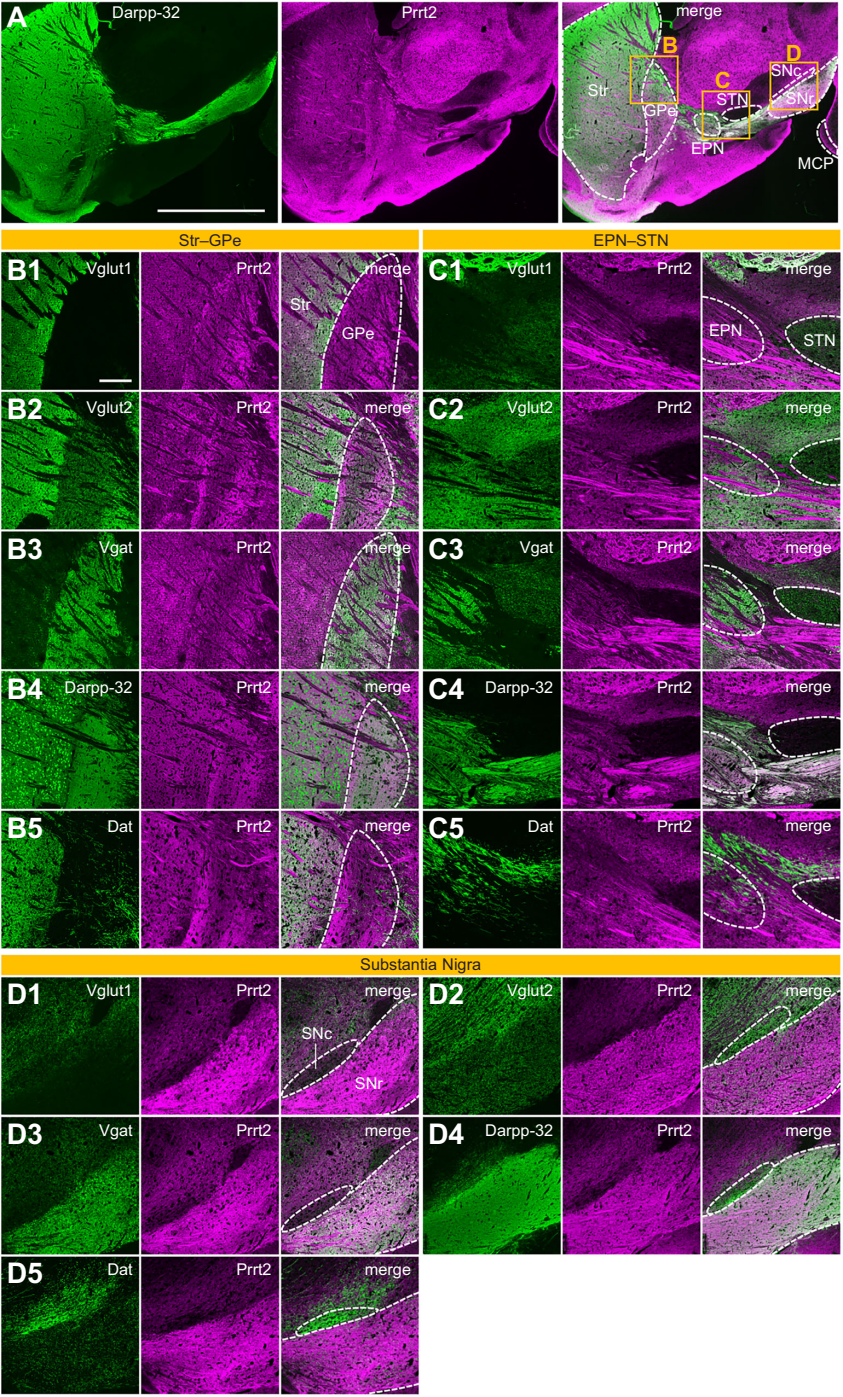

**Figure:**
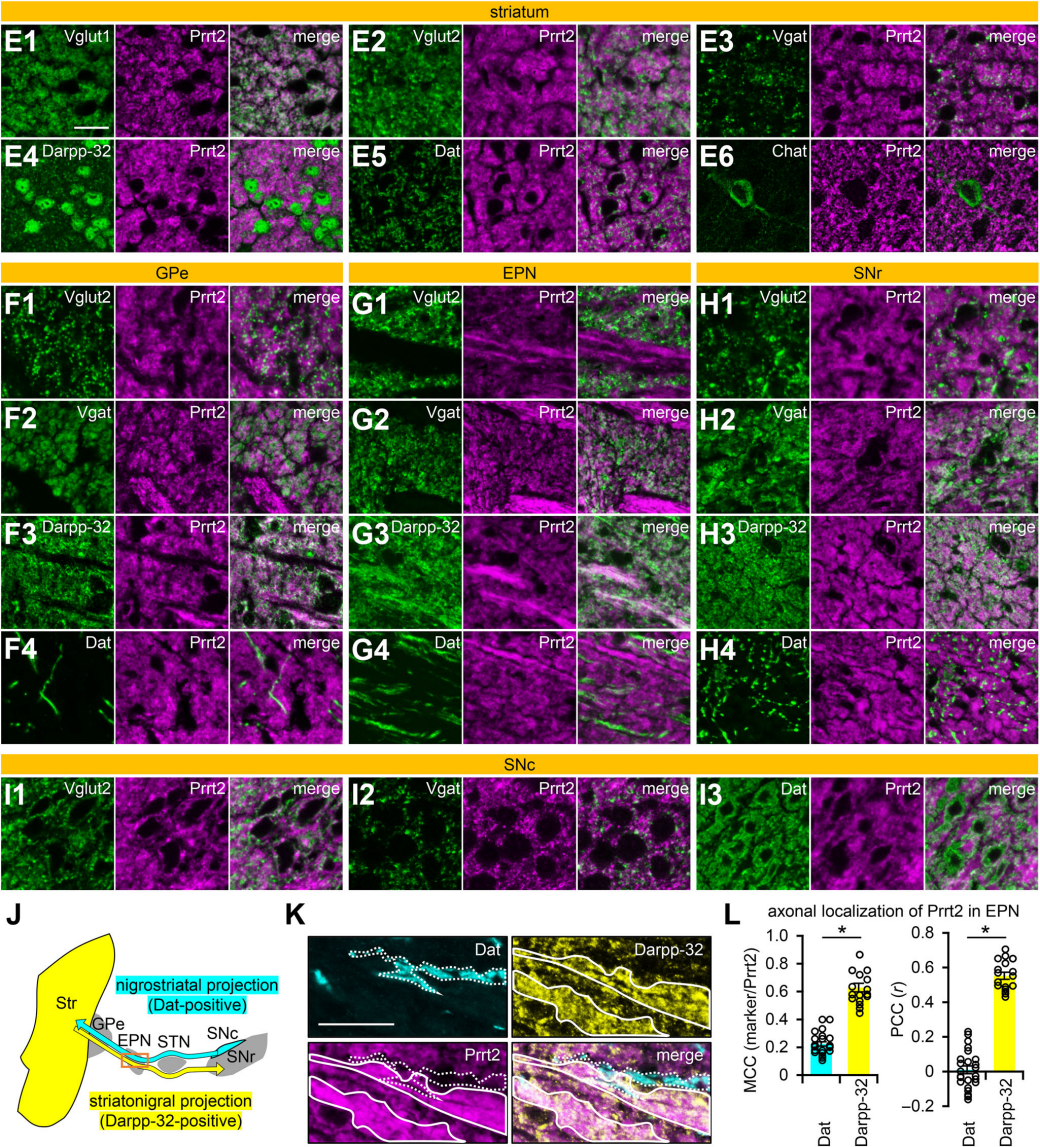

FIGURE 6Quantitative colocalization analysis of Prrt2 in motor‐related brain regions. (A) Representative images used for the colocalization analyses. Wild‐type brain sections were immunostained with anti‐PRRT2 antibodies (magenta) together with antibodies against Vglut1, Vglut2, Vgat, Darpp‐32, or Dat (green). The striatum (Str), external globus pallidus (GPe), entopeduncular nucleus (EPN), substantia nigra pars reticulata (SNr) and compacta (SNc), cerebral cortex (Ctx), thalamus (Thal), pontine nuclei (PN), cerebellar molecular layer (ML), and cerebellar granular layer (GL) were imaged using a confocal microscope with a 100× objective. (B–D) Brain sections of wild‐type mice were co‐immunostained with anti‐Vgat + anti‐Vglut1 antibodies, anti‐Vgat + anti‐Dat antibodies (non‐colocalized controls), or anti‐Stx1 + anti‐Vamp2 antibodies (colocalized control). The striatum was imaged using a confocal microscope with 100× magnification. Scale bar, 20 µm. (E, F) The images were subjected to colocalization analyses, and two parameters were calculated: Manders’ correlation coefficient (MCC, marker/Prrt2) (E) and Pearson's correlation coefficient (PCC) (F). Data are expressed as the mean ± SE and individual value plots (*n* = 4 mice; each individual value represents the mean of six images from 1–3 sections/mouse and 2–6 fields/section). The dashed lines show the mean coefficients of the negative controls, which serve as one of the criteria for colocalization. Two‐way ANOVA of the coefficients showed significant main effects of marker proteins (MCC, *F*_(4, 150)_ = 141.549, *p* < 0.001; PCC, *F*_(4, 150)_ = 146.148, *p* < 0.001) and regions (MCC, *F*_(9, 150)_ = 8.780, *p* < 0.001; PCC, *F*_(9, 150)_ = 20.319, *p* < 0.001), with a significant interaction between the factors (MCC, *F*_(36, 150)_ = 55.900, *p* < 0.001; PCC, *F*_(36, 150)_ = 53.009, *p* < 0.001). Bonferroni post hoc test: Vglut1 versus other markers in the Str, Ctx, Thal, PN, and ML, *p* < 0.001; Vgat or Darpp‐32 versus other markers in the GPe, EPN, and SNr, *p* < 0.001. *p < 0.05.

**Figure:**
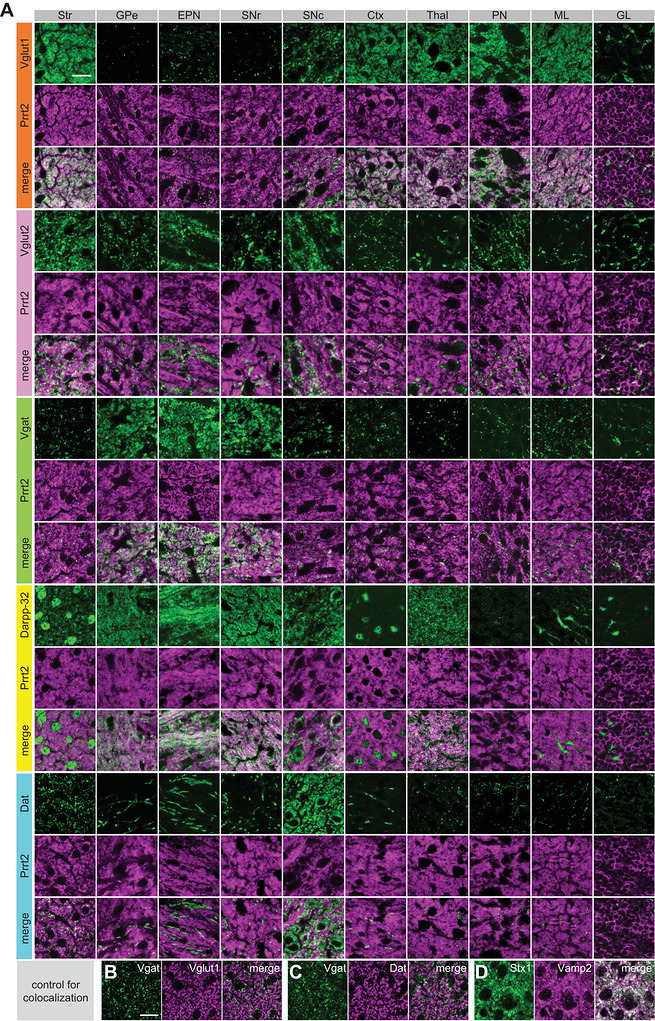

**Figure:**
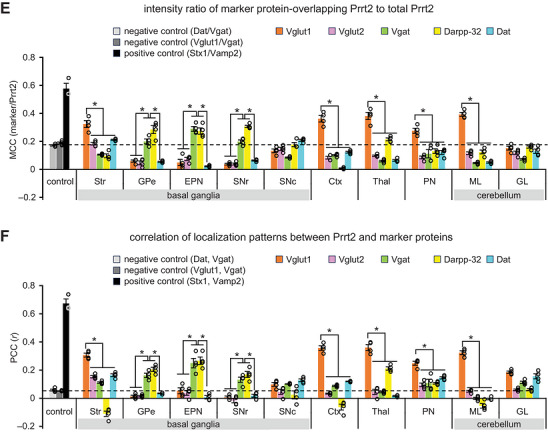

We quantified relative signal intensities of Prrt2 (Figure 3C) in the motor‐related brain regions (Figure 3D). Prrt2 was widespread in the brain, including the cortico‐basal ganglia‐thalamic and cortico‐ponto‐cerebello‐thalamic loops, and was enriched in the cerebellar molecular layer, SNr, and thalamus (Figure 3B,C). Prrt2 levels in the cerebral cortex, pontine nuclei, striatum, GPe, and EPN were also abundant, and those in the SNc and cerebellar granular layer were low to moderate, whereas there were very low Prrt2 immunosignals in the STN, cerebellar Purkinje cell layer, and cerebellar nuclei (Figure 3B,C).

Next, we compared the staining patterns in the whole brains between Prrt2 (Figure 3B) and marker proteins, Vglut1 (Figure 3E), Vglut2 (Figure 3F), Vgat (Figure 3G), Darpp‐32 (Figure 3H), or Dat (Figure 3I). The staining patterns of the marker proteins corresponded with previously reported patterns (Callaerts‐Vegh et al. 2013; Chaudhry et al. 1998; Ehrman et al. 2022; Salahpour et al. 2008). Vglut1 and Vglut2 showed complementary distributions in the brain (Figure 3E,F); Prrt2 showed a Vglut1‐like expression pattern in the cerebral cortex, striatum, thalamus, pontine nuclei, and cerebellum (Figure 3B,E). Among them, all regions except for the cerebellum receive inputs of Vglut1‐positive glutamatergic neurons from the cerebral cortex (Hoerder‐Suabedissen et al. 2018), suggesting that Prrt2 might be expressed in cortical projection neurons. Meanwhile, in most basal ganglia subregions that receive no cortical projections, such as the globus pallidus or substantia nigra, Prrt2 showed an abundant expression pattern similar to that of Vgat (Figure 3G), Darpp‐32 (Figure 3H), and Dat (Figure 3I), but not Vglut1 (Figure 3E). Thus, the neurochemical type of Prrt2‐expressing neurons varies depending on the brain region.

### 3.3 Prrt2 Shows a Vglut1‐Like Subregional Expression Pattern in the Terminal Areas of Cortical Projection Neurons and Cerebellar Granule Cells

In the cerebral cortex, Prrt2 was present in all layers (Figure 4A), whereas Prrt2 levels were somewhat lower in layer IV than in the other layers (Figure 4A). This expression pattern of Prrt2 was similar to that of Vglut1 (Figure 4A1), but not Vglut2 (Figure 4A2) or Vgat (Figure 4A3). As layer IV mainly receives Vglut2‐positive projections from the thalamus (Nahmani and Erisir 2005), Prrt2 might be expressed less in thalamocortical neurons than in neurons that input into the other layers. In high‐magnification imaging using a 100× objective, Prrt2 colocalized with Vglut1 (Figure 4E1) more than Vglut2 (Figure 4E2) or Vgat (Figure 4E3) in the cerebral cortex, suggesting that Prrt2 is partially localized to the Vglut1‐positive synaptic terminals of cortical neurons.

Prrt2 was observed in all thalamic subregions (Figure 4B). Although the ventral lateral nucleus (VL) is one of the motor thalamic nuclei that receives projections from two main motor loops: Vglut2‐positive projections (Figure 4B2) from the cerebellar nuclei (Dacre et al. 2021) and GABAergic (Vgat‐positive) projections (Figure 4B3) from the SNr (Aoki et al. 2019) and the EPN (Miyamoto and Fukuda 2021), Prrt2 levels in the VL were not as high as those in the other thalamic regions. Prrt2 was more abundant in the ventral posteromedial and posterolateral nucleus than in the thalamic reticular nucleus or VL (Figure 4B). This staining pattern was similar to that observed for Vglut1 (Figure 4B1), which was inversely correlated with that of Vglut2 (Figure 4B2). In addition, Prrt2 colocalized with Vglut1 (Figure 4F1), but barely with Vglut2 (Figure 4F2) or Vgat (Figure 4F3) in the thalamus in high‐magnification observations, suggesting that Prrt2 within the thalamus is mainly derived from cortical projections.

Pontine nuclei receive corticopontine projections, mainly consisting of Vglut1‐positive glutamatergic neurons, from layer V of the cerebral cortex (O'Shea et al. 2024). Prrt2 partially overlapped with Vglut1 near the ventral edge of the pontine nuclei, although their staining patterns did not strongly correlate when viewed across the entire pontine nuclei (Figure 4C1). Under high magnification, their colocalization was limited (Figure 4G1). Neither Vglut2 (Figure 4C2,G2) nor Vgat (Figure 4C3,G3) colocalized with Prrt2 in the pontine nuclei under low and high magnifications. Therefore, Prrt2 may be present but not be enriched at any presynaptic terminals in the pontine nuclei. Prrt2 was observed in the longitudinal fascicle of the pons (LFP) (Figure 4C), which is a nerve fiber bundle of corticofugal tracts containing the corticospinal, corticopontine, and corticobulbar projections (Tocco et al. 2022), suggesting that Prrt2 is expressed in at least one of the three cortical projection axons. The axonal localization of Prrt2 was consistent with its expression in the corpus callosum, which consists of cortical axon fibers (Figure 3B), in which no presynaptic markers, Vglut1, Vglut2, or Vgat, were expressed (Figure 3E–G). In addition, Prrt2 was moderately expressed in the middle cerebellar peduncle (Figure 5A), which is an axonal tract of pontocerebellar projection neurons. Therefore, a subset of Prrt2 observed in the pontine nuclei might be postsynaptically expressed in pontocerebellar neurons. A previous in situ hybridization study detected a strong Prrt2 signal in pontine nuclei (Chen et al. 2011), which supports the expression of Prrt2 in pontine neurons.

In the cerebellum, Prrt2 and Vglut1 were both enriched and highly colocalized in the molecular layer (Figure 4D1,H1), in which axons of cerebellar granule cells form parallel fibers and Vglut1‐positive presynaptic terminals connected with the dendrites of the Purkinje cells (Miyazaki et al. 2003). In the granular layer, Prrt2 was observed on the somatic membrane of granule cells to a greater extent than in the neuropil (Figure 4I), whereas Vglut1 (Figure 4I1) and Vglut2 (Figure 4I2) were specifically localized in the neuropil. The Vglut1 and Vglut2 puncta in the granular layer are mainly derived from presynaptic terminals of the mossy fibers (Boulland et al. 2004), which largely originate from pontocerebellar projection neurons. As mentioned above, Prrt2 was detected at low to medium levels in the axons of these neurons (Figure 5A). Mossy fibers also project collateral axons to the cerebellar nuclei; however, Prrt2 immunoreactivity was absent in this region (Figure 4D), suggesting minimal expression of Prrt2 at the presynaptic terminals of pontocerebellar neurons. Thus, Prrt2 appears to be preferentially localized to axons rather than presynaptic terminals in pontocerebellar neurons. Vglut2 in the molecular layer (Figure 4D2,H2) labels climbing fiber terminals (Fremeau et al. 2001), which originate in the inferior olivary nucleus, although Prrt2 and Vglut2 did not colocalize with each other in the molecular layer (Figure 4H2,I2). Vgat was enriched in the cerebellar nuclei (Figure 4D3) because there are presynaptic terminals of GABAergic Purkinje cells; however, Prrt2 was not localized there (Figure 4D). In addition, Prrt2 did not colocalize with Vgat within either the molecular or granular layers (Figure 4H3,I3). Taken together, among cerebellar neurons, Prrt2 was specifically expressed in granule cells, consistent with several previous reports (Michetti et al. 2017; Tan et al. 2018; Calame et al. 2020; Lu et al. 2021).

### 3.4 Prrt2 Shows a Darpp‐32‐Like Expression Pattern in the Basal Ganglia

Darpp‐32 is a marker protein of striatal medium spiny neurons and labels striatal projections to GPe (the indirect pathway), EPN, or SNr (the direct pathways), where Prrt2 was also detected (Figure 5A). To elucidate the type of neurons expressing Prrt2 in each region of the basal ganglia, we performed multiple immunofluorescence staining of Prrt2 and marker proteins, Vglut1, Vglut2, Vgat, Darpp‐32, Dat, or Chat, and imaged the subregions of the basal ganglia using confocal microscopy at 10× (Figure 5B–D) or 100× (Figure 5E–I,K) magnification.

In the striatum, Prrt2 was densely stained in a manner similar to Vglut1 (Figure 5B1) (corticostriatal projection terminals), Vglut2 (Figure 5B2) (thalamostriatal projection terminals), and Dat (Figure 5B5) (nigrostriatal projection terminals and axons) under low magnification; Vglut1 (Figure 5E1) colocalized with Prrt2 more than Vglut2 (Figure 5E2) and Dat (Figure 5E5) under high magnification, suggesting that Prrt2 signals in the striatum were mainly localized in the synaptic terminals of cortical projection neurons, as well as other regions projected from the cerebral cortex (Figure 4E–G). Notably, Prrt2 did not colocalize with Darpp‐32 in the dendrites or soma of medium spiny neurons in the striatum (Figure 5E4). Likewise, Prrt2 did not overlap with a cholinergic neuron marker protein, Chat (Figure 5E6).

Unlike the striatum, the GPe did not receive Vglut1‐positive cortical afferents (Figure 5B1); however, Prrt2 was enriched in the GPe as well as Vgat (Figure 5B3) and Darpp‐32 (Figure 4B4). In high‐magnification observation, Prrt2 was colocalized with Darpp‐32 in the neuropil and striatal axons (Figure 5F3) and overlapped with Vgat in the neuropil, but not in the axons (Figure 5F2), suggesting that Prrt2 was localized at GABAergic presynaptic terminals and axons. *Prrt2* promoter–driven β‐galactosidase staining (Calame et al., 2020) and in situ hybridization analyses (Chen et al. 2011) in previous studies detected Prrt2 signals in the striatum, supporting Prrt2 expression in striatal projection neurons. Vglut2 (Figure 5B2,F1) and Dat (Figure 5B5,F4) were relatively sparse and did not overlap with Prrt2 in the GPe.

In the EPN, there are not only neuropil areas containing synaptic terminals of the direct pathway projections from the striatum, but also axonal tracts of dopaminergic nigrostriatal projections (Figure 5J, cyan) and the direct pathway striatonigral projections (Figure 5J, yellow) in the EPN. Prrt2 was expressed in both the neuropil and the axonal tracts passing through the EPN (Figure 5C). Vglut1 was not detected in the EPN (Figure 5C1), whereas Vglut2 (Figure 5C2) and Vgat (Figure 5C3) were localized exclusively in the neuropil. Under high magnification, Vgat (Figure 5G2), but not Vglut2 (Figure 5G1), was partially colocalized with Prrt2. Dat was localized only in the axons, but did not colocalize with Prrt2 (Figure 4C5,G4). Next, we performed colocalization analysis of Prrt2, Darpp‐32, and Dat (negative control) in the axonal areas (Figure 5K,L). Prrt2 localization highly overlapped (Figure 5L, left, MCC, *p* < 0.05) and correlated (Figure 5L, right, PCC, *p* < 0.05) with that of Darpp‐32 compared to that of Dat, confirming the axonal localization of Prrt2 on the Darpp‐32‐positive striatonigral projections.

The STN receives Vglut1‐positive cortico‐subthalamic hyperdirect pathway projections (Figure 5C1), Vglut2‐positive thalamo‐subthalamic projections (Figure 5C2), and Vgat‐positive external pallido‐subthalamic indirect pathway projections (Figure 5C3). However, Prrt2 was sparsely localized in the STN (Figure 5C), like Darpp‐32 (Figure 5C4) and Dat (Figure 5C5), which is consistent with the lack of a direct connection from the striatum or SNc to the STN. Therefore, Prrt2 is scarcely expressed in these three projections.

Among the two segments of the substantia nigra, Prrt2 was much more abundant in the SNr than in SNc (Figure 5D). The SNr receives Vglut2‐positive subthalamo‐nigral projections (Figure 5D2) (Prasad and Wallén‐Mackenzie 2024), Vgat‐ and Darpp‐32‐positive striatonigral projections (Figure 5D3,D4) (Ehrman et al. 2022), and Dat‐positive dendrites of the SNc neurons (Figure 5D5) (Nirenberg et al. 1996). Under high magnification, Prrt2 partially colocalized with Vgat (Figure 5H2) and Darpp‐32 (Figure 5H3), but not with Vglut2 (Figure 5H1) nor Dat (Figure 5H4) in the SNr. Therefore, Prrt2 in the SNr is derived from striatonigral projection neurons. The SNc contains soma of dopaminergic nigrostriatal projection neurons (Figure 5D5), in which Prrt2 almost did not colocalize with Dat (Figure 5I3), indicating that Prrt2 is poorly expressed in the SNc dopaminergic neurons. Vglut2 (Figure 5I1) or Vgat (Figure 5I2) did not overlap with Prrt2 in the SNc.

### 3.5 Quantitative Colocalization Analysis Supports Region‐Dependent Differences in the Neurochemical Types of Prrt2‐Positive Neurons

Next, we quantified the colocalization of Prrt2 and marker proteins in the images obtained under high magnification (Figure 6), in which two parameters, MCC (Figure 6E; Table 3) and PCC (Figure 6F; Table 4), were calculated. MCC is a simple fraction of overlapping Prrt2 and marker proteins, and PCC represents the correlation between the localization patterns of Prrt2 and marker proteins. We first applied the negative controls, a ratio of Dat to Vgat (Dat/Vgat) (Figure 6B) and a ratio of Vglut1 to Vgat (Vglut1/Vgat) (Figure 6C), and the positive control Stx1/Vamp2 (Figure 6D) into the analysis, validating that these two parameters can be used to evaluate the colocalization. We used the means of the two negative controls as one of the criteria for colocalization (Figure 6E,F, dashed lines). Maker proteins that met the criteria and showed significantly higher values than other marker proteins in both PCC and MCC were considered to colocalize with Prrt2. In the striatum, cerebral cortex, thalamus, pontine nuclei, and cerebellar molecular layer, Vglut1 showed higher colocalization coefficients with Prrt2 in both MCC (Figure 6E) and PCC (Figure 6F) compared to the negative controls and other marker proteins (*p* < 0.05). Similarly, Vgat and Darpp‐32 showed significant colocalization with Prrt2 in the GPe, EPN, and SNr (*p* < 0.05). These quantification results were largely consistent with the observation‐based interpretation (Figures 4E–I, 5E–I, and 6A), confirming Prrt2 expression in the terminal regions of many cortical projection neurons, striatal projection neurons, and cerebellar granule cells. No colocalization coefficients between Prrt2 and each marker protein exceeded those of Stx1a and Vamp2 (positive control) (Figure 6E,F), which may be attributed to widespread subcellular Prrt2 localization not limited to certain zones, such as presynaptic boutons.

**TABLE 3:** Mander's correlation coefficient (marker in Prrt2): Raw data related to Figure 6E.

Vglut1										
	Str	GPe	EPN	SNr	SNc	Ctx	Thal	PN	ML	GL
Mouse #1	0.263	0.036	0.112	0.024	0.097	0.305	0.312	0.239	0.373	0.116
Mouse #2	0.380	0.067	0.012	0.051	0.161	0.345	0.397	0.283	0.370	0.152
Mouse #3	0.313	0.068	0.045	0.039	0.140	0.387	0.386	0.260	0.430	0.182
Mouse #4	0.340	0.068	0.027	0.035	0.132	0.410	0.432	0.323	0.400	0.157
Mean	0.324	0.059	0.049	0.037	0.132	0.362	0.382	0.276	0.393	0.152
SE	0.025	0.008	0.022	0.005	0.013	0.023	0.025	0.018	0.014	0.014

Vglut2										
	Str	GPe	EPN	SNr	SNc	Ctx	Thal	PN	ML	GL
Mouse #1	0.168	0.071	0.088	0.058	0.118	0.102	0.095	0.058	0.092	0.086
Mouse #2	0.165	0.067	0.041	0.035	0.153	0.101	0.093	0.089	0.134	0.130
Mouse #3	0.206	0.012	0.085	0.027	0.139	0.065	0.090	0.091	0.126	0.140
Mouse #4	0.195	0.036	0.081	0.032	0.173	0.066	0.112	0.104	0.109	0.118
Mean	0.184	0.046	0.074	0.038	0.146	0.084	0.097	0.085	0.115	0.118
SE	0.010	0.014	0.011	0.007	0.011	0.010	0.005	0.010	0.009	0.012

Vgat										
	Str	GPe	EPN	SNr	SNc	Ctx	Thal	PN	ML	GL
Mouse #1	0.099	0.162	0.293	0.215	0.079	0.087	0.073	0.063	0.051	0.083
Mouse #2	0.103	0.187	0.262	0.190	0.077	0.111	0.065	0.150	0.054	0.069
Mouse #3	0.113	0.244	0.268	0.220	0.089	0.106	0.044	0.060	0.035	0.058
Mouse #4	0.108	0.207	0.335	0.167	0.093	0.102	0.056	0.173	0.042	0.081
Mean	0.106	0.200	0.289	0.198	0.085	0.101	0.060	0.112	0.045	0.072
SE	0.003	0.017	0.017	0.012	0.004	0.005	0.006	0.029	0.004	0.006

Darpp‐32										
	Str	GPe	EPN	SNr	SNc	Ctx	Thal	PN	ML	GL
Mouse #1	0.086	0.205	0.245	0.302	0.164	0.001	0.225	0.177	0.127	0.162
Mouse #2	0.074	0.316	0.334	0.331	0.162	0.015	0.195	0.097	0.129	0.175
Mouse #3	0.089	0.331	0.282	0.299	0.221	0.005	0.238	0.127	0.153	0.137
Mouse #4	0.138	0.286	0.234	0.295	0.161	0.007	0.199	0.119	0.087	0.166
Mean	0.097	0.285	0.274	0.307	0.177	0.007	0.214	0.130	0.124	0.160
SE	0.014	0.028	0.022	0.008	0.015	0.003	0.010	0.017	0.014	0.008

Dat										
	Str	GPe	EPN	SNr	SNc	Ctx	Thal	PN	ML	GL
Mouse #1	0.206	0.046	0.020	0.053	0.220	0.136	0.055	0.079	0.039	0.107
Mouse #2	0.216	0.047	0.023	0.064	0.212	0.126	0.058	0.130	0.044	0.101
Mouse #3	0.202	0.055	0.031	0.070	0.188	0.118	0.082	0.141	0.054	0.170
Mouse #4	0.219	0.063	0.015	0.065	0.209	0.109	0.062	0.133	0.061	0.141
Mean	0.211	0.053	0.022	0.063	0.207	0.122	0.064	0.121	0.049	0.129
SE	0.004	0.004	0.003	0.004	0.007	0.006	0.006	0.014	0.005	0.016

**TABLE 4:** Pearson's correlation coefficient between Prrt2 and marker proteins: Raw data related to Figure 6F.

Vglut1										
	Str	GPe	EPN	SNr	SNc	Ctx	Thal	PN	ML	GL
Mouse #1	0.279	−0.005	0.068	0.011	0.062	0.317	0.314	0.259	0.316	0.191
Mouse #2	0.335	0.004	0.026	0.027	0.116	0.351	0.338	0.261	0.289	0.175
Mouse #3	0.286	0.034	0.104	−0.012	0.093	0.364	0.392	0.207	0.350	0.206
Mouse #4	0.327	0.018	0.014	0.005	0.124	0.395	0.398	0.267	0.340	0.169
Mean	0.307	0.013	0.053	0.008	0.099	0.357	0.360	0.248	0.324	0.185
SE	0.014	0.008	0.021	0.008	0.014	0.016	0.021	0.014	0.014	0.008

Vglut2										
	Str	GPe	EPN	SNr	SNc	Ctx	Thal	PN	ML	GL
Mouse #1	0.138	0.005	0.047	0.004	0.068	0.037	0.068	0.069	0.032	0.048
Mouse #2	0.175	0.017	0.067	0.012	0.072	0.033	0.072	0.129	0.052	0.058
Mouse #3	0.144	0.010	0.052	−0.044	−0.006	0.023	−0.006	0.101	0.056	0.075
Mouse #4	0.154	0.027	−0.011	−0.041	0.046	0.039	0.046	0.082	0.059	0.061
Mean	0.153	0.015	0.038	−0.017	0.045	0.033	0.045	0.095	0.050	0.060
SE	0.008	0.005	0.017	0.015	0.018	0.004	0.018	0.013	0.006	0.005

Vgat										
	Str	GPe	EPN	SNr	SNc	Ctx	Thal	PN	ML	GL
Mouse #1	0.108	0.128	0.238	0.164	0.100	0.077	0.041	0.070	0.016	0.135
Mouse #2	0.101	0.149	0.251	0.144	0.101	0.086	0.032	0.062	−0.007	0.105
Mouse #3	0.125	0.200	0.173	0.140	0.107	0.090	0.054	0.124	0.005	0.097
Mouse #4	0.110	0.173	0.336	0.079	0.098	0.096	0.049	0.176	−0.020	0.112
Mean	0.111	0.162	0.249	0.132	0.101	0.087	0.044	0.108	−0.002	0.112
SE	0.005	0.015	0.033	0.018	0.002	0.004	0.005	0.027	0.008	0.008

Darpp‐32										
	Str	GPe	EPN	SNr	SNc	Ctx	Thal	PN	ML	GL
Mouse #1	−0.087	0.194	0.262	0.222	0.050	−0.123	0.242	0.120	−0.082	0.056
Mouse #2	−0.164	0.237	0.337	0.163	−0.045	−0.038	0.188	0.100	−0.053	0.075
Mouse #3	−0.109	0.220	0.254	0.173	0.085	−0.056	0.218	0.112	−0.008	0.048
Mouse #4	−0.081	0.168	0.212	0.118	0.005	−0.033	0.199	0.094	−0.070	0.073
Mean	−0.110	0.205	0.266	0.169	0.024	−0.062	0.212	0.106	−0.053	0.063
SE	0.019	0.015	0.026	0.021	0.028	0.021	0.012	0.006	0.016	0.007

Dat										
	Str	GPe	EPN	SNr	SNc	Ctx	Thal	PN	ML	GL
Mouse #1	0.162	0.037	0.035	0.036	0.150	0.122	0.018	0.129	−0.011	0.139
Mouse #2	0.184	0.038	0.018	0.033	0.138	0.122	0.019	0.154	−0.011	0.116
Mouse #3	0.137	0.027	−0.011	−0.010	0.106	0.113	0.007	0.163	−0.010	0.197
Mouse #4	0.170	0.038	−0.014	0.011	0.114	0.120	0.021	0.133	0.013	0.171
Mean	0.163	0.035	0.007	0.018	0.127	0.119	0.016	0.145	−0.005	0.156
SE	0.010	0.003	0.012	0.011	0.010	0.002	0.003	0.008	0.006	0.018

### 3.6 Prrt2 Is Localized at Both D1‐Specific and D2‐Specific Regions in the Striatum

**FIGURE 7:**
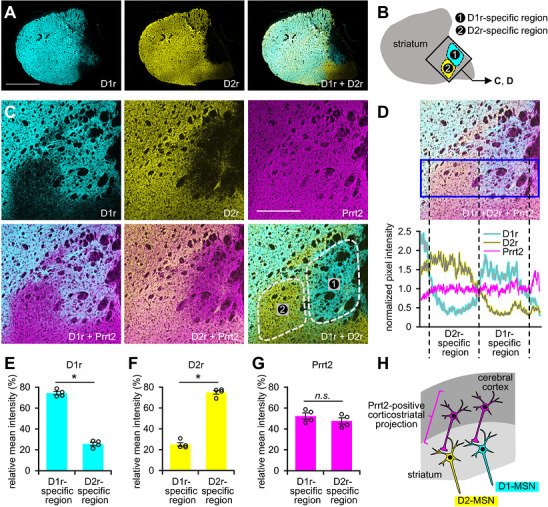
Prrt2 localization in the D1r‐specific and D2r‐specific regions of the striatum. (A) Sagittal sections of wild‐type mice were co‐immunostained with anti‐D1r and anti‐D2r antibodies, and the lateral striata were imaged using a fluorescence microscope at a low magnification. (B) Positions of the D1r‐ or D2r‐specific regions in the lateral striatum are depicted. The box shows the imaged area at a higher magnification in the following imaging. (C) Sagittal sections were co‐immunostained with anti‐D1r, anti‐D2r, and anti‐Prrt2 antibodies, and the lateral striata were imaged using a confocal microscope with a 10× objective. (D) Normalized pixel intensities of D1r, D2r, and Prrt2 within the boxed area in the upper panel are shown in the lower panel. The dotted lines represent the rostral and caudal edges of the D1r‐ and/or D2r‐specific regions. (E) Relative mean intensities of D1r (left), D2r (center), and Prrt2 (right) in the D1r‐ or D2r‐specific regions are shown. Student's *t*‐test: *t*_(6)_ = 20.669, *p* < 0.001 (D1r, left); *t*_(6)_ = −17.916, *p* < 0.001 (D2r, center); *t*_(6)_ = 1.097, *p* = 0.315 (Prrt2, right). *p < 0.05. (F) Schematic showing that Prrt2 is expressed in corticostriatal neurons projecting to both the D1r‐ and D2r‐positive medium spiny neurons (D1‐ and D2‐MSN, respectively). Scale bars: 1 mm (A) and 400 µm (C).

In the striatum, Prrt2 was mainly localized at the Vglut1‐positive presynaptic terminals of corticostriatal projection neurons (Figures 5E1 and 6E,F). Two types of striatal medium spiny neurons receive the corticostriatal projections: D1r‐positive direct pathway and D2r‐positive indirect pathway neurons. Meanwhile, considering that corticostriatal (Figure 5E1) and corticothalamic (Figure 4F1) neurons expressed Prrt2 at higher levels or frequencies than cortico‐subthalamic (the hyperdirect pathway) (Figure 5C) or corticopontine (Figure 4G1) neurons, Prrt2 expression levels were not equivalent among the types of cortical projection neurons; therefore, we quantified and compared expression levels of Prrt2 in the two types of corticostriatal neurons (Figure 7). Although the two types of striatal neurons are equivalently intermingled in most spaces of the striatum, a recent study has reported the presence of D1r‐specific or D2r‐specific zones in the caudal striatum (Figure 7A,B) (Ogata et al. 2022). In triple immunofluorescence staining of D1r, D2r, and Prrt2 (Figure 7C,D), we first confirmed the selective localization of D1r (Figure 7E) and D2r (Figure 7F) to their specific zones (*p* < 0.05). We then found that Prrt2 was almost equally localized in the D1r‐specific and D2r‐specific zones (Figure 7D,G) (*p* = 0.315), indicating that Prrt2 was similarly expressed in the two types of corticostriatal projection neurons connected to the direct and indirect pathway striatal neurons, respectively (Figure 7H).

## 4 Discussion

### 4.1 Comparison of Prrt2 Localization in the Mouse Brain and Primary Midbrain Neurons

The present study revealed that Prrt2 is preferentially expressed in excitatory and inhibitory neurons rather than dopaminergic neurons in both the primary midbrain culture (Figure 2) and the mouse brain (Figures 3, 4, 5, 6, 7). Prrt2 expression in the excitatory and inhibitory neurons was consistent with a previous study in hippocampal cultures (Valente et al. 2019). Prrt2 was expressed in Vglut1‐ and Vglut2‐positive neurons to the same extent in the primary culture (Figure 2H), but more abundantly in Vglut1‐positive neurons than in Vglut2‐positive neurons in the mouse brain (Figure 6). This might be attributed to subtype switching from Vglut2 to Vglut1 during brain development (Miyazaki et al. 2003). Poor expression of Prrt2 in dopaminergic neurons was consistently observed both in primary cultures (Dat, Th; Figure 2B,H) and in the adult brain (Dat; Figures 5E5,F4,G4,H4,I3 and 6E,F). Although primary cultures of cholinergic neurons were not prepared in this study, Prrt2 expression was clearly segregated from the cholinergic marker Chat in the mouse brain (Figure 5E6). Moreover, Prrt2 was localized not only at presynaptic terminals but also widely in axons, perisynaptic areas, and somatic plasma membranes in the primary midbrain neurons (Figure 2), which is consistent with a previous study in hippocampal neurons (Sterlini et al. 2021), and was also identical to its subcellular localization in the brain; Prrt2 was localized in the axonal tracts of cortical (Figure 3B, the corpus callosum), striatal (Figure 5K, striatonigral tract), and pontine (Figure 5A, the middle cerebellar peduncle) neurons and the somatic membrane of cerebellar granule cells (Figure 4I) and at or near the presynaptic sites of the corticostriatal (Figure 5E1), corticothalamic (Figure 4F1), corticocortical (Figure 4E1), corticopontine (Figure 4G1), striatopallidal (Figure 5F2), striato‐entopedunclar (Figure 5G2), and striatonigral (Figure 5H2) neurons and cerebellar granule cells (Figure 4H1, in the molecular layer).

Notably, Prrt2 expression and subcellular localization may be dynamically regulated by neuronal activity via activity‐dependent processing, such as proteolytic cleavage (Hatta et al. 2020). As this study was based on steady‐state tissue analysis, it may not fully capture the dynamic changes in Prrt2 distribution. Future investigations employing activity‐inducing models, such as KCl stimulation, will be valuable for a more comprehensive understanding of Prrt2 dynamics.

### 4.2 Prrt2 Roles in the Cortico‐Ponto‐Cerebello‐Thalamic Loop

**FIGURE 8:**
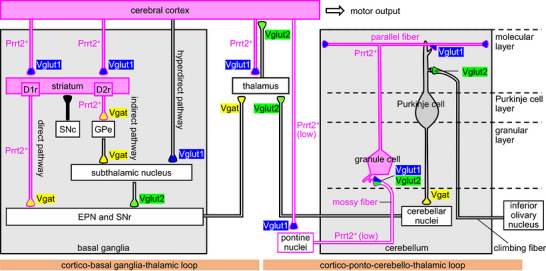
**S**chematic representation of Prrt2 localization in the two main motor loops. In the schematic of the two main motor loops, Prrt2‐expressing neurons are highlighted with magenta (the filled, high expression; the open, low expression). Vglut1‐, Vglut2‐, or Vgat‐positive presynaptic terminals are highlighted with blue, green, and yellow, respectively. Prrt2 is expressed both in glutamatergic (Vglut1‐positive) and GABAergic (Vgat‐positive) neurons in the loops. In the cortico‐basal ganglia‐thalamic loop (left), Prrt2 is expressed in the corticostriatal (from the cerebral cortex to the striatum), striato‐entopedunclar, striatonigral (the D1r‐positive direct pathways) (from the striatum to the EPN and SNr, respectively), and striatopallidal (the D2r‐positive indirect pathway) (from the striatum to the GPe) projections, although the latter of the indirect pathways constituted by the pallido‐subthalamic (from the GPe to the STN) and subthalamo‐nigral projections (from the STN to the SNr) show almost no expression of Prrt2. The nigrostriatal (from the SNc to striatum, dopaminergic), nigrothalamic (from the SNr to the thalamus), and thalamo‐cortical (from the thalamus to the cerebral cortex) projections sparsely express Prrt2; however, cortico‐thalamic projections highly express Prrt2. In the cortico‐ponto‐cerebello‐thalamic loop (right), Prrt2 is highly expressed in the cerebellar granule cells and partially expressed in corticopontine (from the cerebral cortex to pontine nuclei) and ponto‐cerebellar (from the pontine nuclei to the cerebellar granular layer) (a main component of the mossy fiber) projections. Prrt2 is not expressed in the cerebello‐thalamic projections (from the cerebellar nuclei to the thalamus) or climbing fiber (from the inferior olivary nucleus to the cerebellar molecular layer). GPe, external globus pallidus; EPN, entopeduncular nucleus; STN, subthalamic nucleus; SNc, substantia nigra pars compacta; SNr, substantia nigra pars reticulata.

In this study, we revealed that Prrt2 is expressed in two main motor‐related circuits (Figures 3, 4, 5, 6, 7). In the cortico‐ponto‐cerebello‐thalamic loop (Figure 8, right), Prrt2 was selectively expressed in glutamatergic neurons (Figure 4), unlike in the cortico‐basal‐ganglia‐thalamic loop (Figure 5). Cerebellar granule cells were most abundantly expressing Prrt2 in the brain (Figures 3C and 4D1,H1). Thus, the present study emphasizes the importance of cerebellar granule cells in physiological Prrt2 roles, supporting a previous study (Tan et al. 2018), which showed that cerebellar granule cell‐specific Prrt2 deletion results in PKD‐like behavioral phenotypes in a manner similar to systemic Prrt2 deletion. In addition, we found that corticopontine (Figure 4C1,G1) and pontocerebellar neurons (Figures 4D1,D2,I1,I2 and 5A) were also but limitedly expressing Prrt2. The neurons are located upstream of the cerebellum in the loop (Figure 8, right); therefore, Prrt2 could regulate both the input and output signals of the cerebellar granule cells.

### 4.3 Prrt2 Roles in the Cortico‐Basal Ganglia‐Thalamic Loop

In the cortico‐basal ganglia‐thalamic loop (Figure 8, left), Prrt2 was mainly localized at and around the glutamatergic terminals that provide input from the cerebral cortex to the striatum, and the GABAergic terminals that provide output from the striatum. Since Prrt2 is known to negatively regulate neuronal excitability (Fruscione et al. 2018) and neurotransmitter release (Coleman et al. 2018), striatum‐centered Prrt2 localization might contribute to the activity control of the striatum and downstream brain regions, such as the GPe, EPN, and SNr. The direct and indirect pathways pass through the striatum and are related to motor promotion and suppression, respectively (Chiken et al. 2015), suggesting that Prrt2 could be implicated in both movement promotion and suppression. We previously reported that Prrt2 regulates activity‐dependent dopamine release in the striatum (Hatta et al. 2024). However, in this study, we found poor Prrt2 expression in dopaminergic neurons (Figures 2B and 5I3). Considering that Prrt2 deficiency elevates glutamate levels in the cerebral cortex (Mo et al. 2019), Prrt2 may control glutamate release from corticostriatal presynaptic terminals and subsequent glutamate‐induced dopamine release (Krebs et al. 1991; Moghaddam et al. 1990) or synaptic plasticity. A recent study showed that enhanced dopamine fluctuations form dyskinesia‐related synaptic plasticity in the GABAergic presynaptic terminals of striatal projection neurons (Abe et al. 2023), implying that Prrt2 deficiency‐induced enhancement of dopamine release might similarly cause abnormal plasticity.

Although this study primarily focused on the role of PRRT2 in motor circuits, it is worth noting that PRRT2 is also a causative gene for benign familial infantile epilepsy (Ono et al. 2012). Epileptic seizures generally arise in various brain regions, such as the hippocampus, cerebral cortex, and thalamus, depending on their type and etiology. The basal ganglia also contribute to seizure control; in particular, corticostriatal projection neurons (Miyamoto et al. 2019) and striatal neurons (Hyder et al. 2025), both of which express Prrt2, have been shown to modulate seizure activity. These findings raise the possibility that Prrt2 plays a critical role not only in excitatory neurons but also in inhibitory neurons involved in the regulation of seizure susceptibility.

## 5 Conclusion

Although Prrt2 was distributed throughout the mouse brain as previously observed, we found that it was preferentially expressed in Vglut1‐positive glutamatergic neurons and GABAergic neurons, rather than in dopaminergic or cholinergic neurons. Moreover, Prrt2 exhibited a circuit‐dependent expression pattern: It was expressed in excitatory neurons in the cerebral cortex and cerebellum, whereas inhibitory neurons predominantly expressed Prrt2 in the basal ganglia. Although a detailed analysis of the pathophysiological roles of Prrt2 in inhibitory neurons that have not been characterized as much as those in excitatory neurons is required, our findings indicate that the basal ganglia might represent another key region where Prrt2 plays a role in the control of motor function and seizure susceptibility. In addition, our observation that Prrt2 is localized at subregional or subcellular areas responsible for fine‐tuning circuit excitability within the basal ganglia and cerebellum corroborates the role of Prrt2 in the suppression of neuronal activity at presynaptic sites or axons and supports the interpretation of the neurochemical or behavioral phenotypes of *Prrt2* mutant mice, as previously reported by our group and other research groups.

Thus, our findings will provide critical information for future studies on Prrt2 functions, molecular mechanisms of dyskinesia and related disorders, and the regulation of neural circuit excitability.

## Author Contributions

D.H. and K.W. performed experiments and data analysis. D.H. and N.I. wrote the manuscript. D.H., K.W., A.K., K.Y., N.K., K.S., and N.I. designed this study and discussed the results. All authors have read and approved the final manuscript.

## Funding

This work was supported in part by funds from the Program for Intractable Diseases Research (JP16ek0109119h), the Japan Agency for Medical Research and Development (AMED) to N.I., K.S., and N.K., and JSPS KAKENHI Grants 18H02720, 21H02809, and 25K02416 to N.I. and grants 20K22688 and 25K19033 to D.H.

## Ethics Statement

The animal study was approved by the Institutional Animal Care and Use Committee of Nagasaki University.

## Conflicts of Interest

The authors declare no conflicts of interest.

## Data Availability

The data that support the findings of this study are available from the corresponding author upon reasonable request.
